# Japanese Encephalitis, Singapore

**DOI:** 10.3201/eid1203.051251

**Published:** 2006-03

**Authors:** Yin-Ling Koh, Boon-Huan Tan, Jimmy Jin-Phang Loh, Eng-Eong Ooi, Sheng-Yong Su, Li-Yang Hsu

**Affiliations:** * Singapore General Hospital, Singapore;; † Defense Science Organization, Singapore;; ‡ National University of Singapore, Singapore

**Keywords:** Japanese encephalitis, Singapore, encephalitis virus, Japanese, letter

**To the Editor:** Japanese encephalitis (JE) is an endemic flavivirus disease in Asia. The JE virus (JEV) is one of the leading causes of viral encephalitis: 35,000–50,000 cases occur every year (*1*). While most infections are subclinical, the disease has a high case-fatality rate (≈25%) and considerable incidence of serious neurologic sequelae with the development of overt meningoencephalitis (*1*).

JEV is transmitted principally by *Culex tritaeniorhynchus* and less frequently by *Cx. vishnui* and *Cx. gelidus*, which breed in flooded rice fields. The virus circulates in waterfowl such as herons and egrets, and pigs serve as amplifying hosts. Hence, the distribution of JEV is significantly linked to irrigated rice production and pig rearing (*2*).

JEV was previously endemic in Singapore, but since the phasing out of pig farming (completed in 1992), the incidence of reported disease has become very low. Routine serologic testing for JEV has correspondingly been dropped from local hospital microbiology laboratories. We describe an indigenous case of JEV meningoencephalitis in Singapore.

In May 2005, a 53-year-old previously healthy man of Chinese ethnicity was seen at Singapore General Hospital with a 1-week history of fever and abdominal pain. Altered mental status had developed shortly after the onset of fever. He had worked in the western part of Singapore as a lifeguard at a community swimming pool and had not traveled, even to offshore islands, for the past year.

On examination, he was febrile with a temperature of 39.3°C and disoriented to time and place. Nuchal rigidity was present, and hyperreflexia was demonstrated in both upper limbs, although lower limb reflexes were normal. The rest of the initial physical examination was unremarkable.

Laboratory studies showed a leukocyte count of 4.91 × 10^9^/L, hemoglobin concentration of 14.3 g/dL, and platelet count of 171 × 10^9^/L. Serum and liver biochemistry results were normal. Magnetic resonance imaging of the brain showed mild leptomeningeal enhancement. An electroencephalogram showed generalized slow waves, consistent with severe diffuse encephalopathy. A lumbar puncture was performed. The opening pressure was elevated at 24 cm/H_2_O; cerebrospinal fluid (CSF) leukocyte count was 192/mm^3^, consisting mostly of lymphocytes; CSF glucose was 2.4 mmol/L (44% of serum glucose concentration); and CSF total protein was elevated at 1.5 g/L. CSF and blood cultures for bacteria, fungi, and mycobacteria were negative, as were CSF isolates for enteroviruses and herpes simplex virus.

Results of paired acute- and convalescent-phase serologic testing for dengue immunoglobulin M (IgM) and IgG were negative, as were the microscopic agglutination test for leptospirosis and the Widal test for typhoid. Subsequent polymerase chain reaction (PCR) testing of serum and CSF on day 10 of illness yielded negative results for Nipah/Hendra virus, West Nile virus, enterovirus, herpesviruses, measles virus, and alphaviruses.

However, the patient's serum but not CSF tested positive for flavivirus RNA when a universal flavivirus reverse transcription (RT)–PCR assay that targets the conserved sequence of the NS5 region was used (*3*). JEV was definitively identified as the etiologic agent when the serum sample tested positive with a second RT-PCR specific to the conserved sequences in the NS3 region of the JEV genome, modified to a real-time platform (*4*). Comparison of the 197-nt sequence of this JEV-specific RT-PCR product with the library of human, mouse, and viral genome databases managed by the National Center for Biotechnology Information site using the BLASTN program (available from http://www.abcc.ncifcrf.gov/app/htdocs/appdb/appinfo.php?appname) showed 93% homology with reported JEV sequences.

The patient had a prolonged and complicated hospital stay. He became comatose and went into type 2 respiratory failure within 72 hours of hospitalization; pinpoint pupils, bradycardia, and hypothermia developed. These developments necessitated mechanical ventilation at the medical intensive care unit, where the patient subsequently improved after 6 days of supportive care and was extubated. Flaccid paraparesis with urinary retention developed at this point, and magnetic resonance imaging of the spine demonstrated signal enhancement at the level of the conus medullaris. Motor power gradually improved with intensive rehabilitation and was normal by the time of the patient's discharge 2 months after admission. However, intermittent self-catheterization was still required for detrusor hyperreflexia.

This is the sixth case of JE reported in Singapore from 1991 to July 2005. Three imported cases were reported from 1991 to 2000. Two patients whose cases were reported in 2001 had no substantial travel history and likely acquired the infection within Singapore, as our patient did. However, the lack of diagnostic testing by local service microbiology laboratories has possibly led to underdiagnoses of this disease.

While abolishment of pig farming in Singapore has greatly reduced the risk for epidemic transmission of JEV, a seroepidemiologic study on the prevalence of neutralizing antibodies to JEV in local animals (including dogs, cattle, goats, imported pigs, chickens, and crows) showed a JEV antibody prevalence of 46.5% in working dogs and 60% in chickens. These findings suggest that JEV remains active in Singapore (*5*). The virus reservoir is likely to be aquatic birds. The threat of JE remains, and public health vigilance for this vectorborne disease should not diminish.

The infrequent incidence of JE in Singapore is insufficient to justify routine vaccination for travelers to this country. However, JE remains a rare differential diagnosis for travelers from or passing through Singapore.
